# The CRISPR/Cas9-Mediated Knockout of *VgrG2* in Wild Pathogenic *E. coli* to Alleviate the Effects on Cell Damage and Autophagy

**DOI:** 10.3390/vetsci12030249

**Published:** 2025-03-05

**Authors:** Tian-Ling Pan, Jin-Long Cha, Hao Wang, Jing-Song Zhang, Jin-Long Xiao, Jue Shen, Meng Zhou, Yue Li, Jin-Zhi Ma, Kai-Yuan Zhao, Yong-Kang Zhang, Peng Xiao, Hong Gao

**Affiliations:** 1 College of Veterinary Medicine, Yunnan Agricultural University, Kunming 650201, China; 13759420973@163.com (T.-L.P.); 18287258920@163.com (J.-L.C.); zhangjs228612@163.com (J.-S.Z.); xiaojl_h@163.com (J.-L.X.); nicetomeet_jue@163.com (J.S.); 18184868232@163.com (Y.L.); 15108713682@163.com (J.-Z.M.); z17716069408@163.com (K.-Y.Z.); zhangyongkang0313@163.com (Y.-K.Z.); 2 College of Food Science and Technology, Yunnan Agricultural University, Kunming 650201, China; wanghaoxu0117@163.com (H.W.); zhoumeng@mail.kiz.ac.cn (M.Z.)

**Keywords:** wild-type *E. coli*, CRISPR/Cas9, *VgrG2* gene, large fragment deletion, mTOR signaling pathway

## Abstract

CRISPR/Cas9, as a well-established gene editing technology, has been applied in numerous model organisms, but its application in wild-type *E. coli* remains limited. Pathogenic wild-type *E. coli*, a major cause of foodborne illnesses and intestinal inflammation in humans and animals, poses a significant global public health threat. The valine-glycine repeat protein G (VgrG) is a key virulence factor that enhances *E. coli* pathogenicity. In this study, PCR was used to identify 50 strains carrying the virulence gene *VgrG2* out of 83 wild pathogenic *E. coli* strains, with only one strain sensitive to kanamycin and spectinomycin. A homologous repair template for *VgrG2* was constructed using overlap PCR. A dual-plasmid CRISPR/Cas9 system, combining pTarget (spectinomycin resistance) and pCas (kanamycin resistance) with Red homologous recombination, was then used to induce genomic cleavage and knock out *VgrG2*. PCR and sequencing confirmed the deletion of a 1708 bp fragment of the *VgrG2* gene in wild-type *E. coli*. IPEC-J2 cells were infected with *E. coli*-WT and *E. coli* ∆*VgrG2*, and treated with the mTOR inhibitor rapamycin to study the effects of *VgrG2* on the mTOR signaling pathway. The qPCR results showed that *VgrG2* activated the mTOR pathway, suppressed *mTOR* and *p62* mRNA levels, and upregulated the autophagy-related genes and LC3-II protein expression. In conclusion, we utilized CRISPR/Cas9 technology to achieve large-fragment deletions in wild-type *E. coli*, revealing that *VgrG2* activates the mTOR signaling pathway and upregulates autophagy markers. These findings offer new insights into *E. coli* genome editing and clarifies the pathogenic mechanisms through which *VgrG2* induces cellular damage.

## Simple Summary

This study focuses on the efficacy of CRISPR/Cas9 technology in wild-type *E. coli*, a major pathogen responsible for foodborne illnesses and intestinal inflammation. The primary objective was to investigate the role of *VgrG2*, a key virulence factor, in enhancing *E. coli* pathogenicity. We screened *E. coli* strains harboring the *VgrG2* gene, which were also susceptible to both kanamycin and spectinomycin, and successfully knocked out the *VgrG2* gene using a dual-plasmid CRISPR/Cas9 system. The results showed that *VgrG2* activated the mTOR signaling pathway, inhibited *mTOR* and *p62* mRNA levels, upregulated autophagy-related genes, and increased the expression of the LC3-II protein. These findings enhance our understanding of the molecular mechanisms by which *VgrG2* contributes to cellular damage and provide valuable insights into genome editing strategies for pathogenic bacteria, offering potential approaches for controlling bacterial infections and their associated health impacts.

## 1. Introduction

The CRISPR/Cas9 system serves as an immune defense mechanism that originated in prokaryotic organisms [1,2], composed of CRISPR sequences and Cas9 proteins [3,4]. It functions by recognizing foreign DNA sequences and incorporating them into the CRISPR spacer. The Cas9 nuclease identifies protospacer adjacent motifs (PAM) on foreign DNA and cleaves the exogenous DNA, thus protecting the host cell [5,6]. This system offers a greater precision, simplicity, and efficiency compared to traditional gene editing systems like TALEN and ZFN [7,8]. When applied for gene editing, the CRISPR/Cas9 system must be expressed in the target cells to produce the Cas9 protein and guide RNA (gRNA). The gRNA then directs Cas9 to recognize the target DNA sequence and the PAM (Protospacer Adjacent Motif) sequence, enabling the cleavage of the target DNA and generating a double-stranded break (DSB). When a double-strand break (DSB) occurs, cells initiate self-repair mechanisms to prevent death, with homologous recombination repair (HDR) being one of the key pathways for DNA repair. In the presence of homologous sequences, cells utilize the HDR mechanism for DSB repair. However, the HDR system is primarily found in eukaryotes and is largely absent in prokaryotes, which limits the application of this technology in wild-type *E. coli* [7]. Therefore, when using this system for gene editing, an HDR template must be added to the vector to facilitate the repair of broken DNA. Jiang et al. constructed a two-plasmid system that incorporated a homologous recombination mechanism. They co-transfected the donor sequence along with the construction plasmid into *E. coli* MG1655, successfully achieving gene knockout in this strain using the CRISPR/Cas9 system [9]. With this protocol, the CRISPR/Cas9 system enables the precise and efficient rejection of specific target genes within target strains. The co-transformation strategy of the donor sequence and plasmid not only optimizes the protocol, but also significantly reduces the complexity of the technical operation. Similarly, Wang et al. successfully induced site-directed mutations of the proB gene by using pCas9 and pTargetF+ vectors in Escherichia coli BL21. This method significantly reduces the likelihood of the repeat cleavage of gRNA/Cas9 and preserves the integrity of the PAM sequence, preventing the introduction of unnecessary silencing mutations in the genome [10]. At present, gene editing research in genetically engineered bacteria is relatively well established, but the research in wild-type *E. coli* has rarely been reported. In particular, research focusing on the deletion of large fragments of the virulence genes in wild-type *E. coli* is even more limited.

The Type VI Secretion System (T6SS) was first identified in Pseudomonas aeruginosa, and the presence of this secretory system has subsequently been observed in some gram-negative bacteria. A key component of T6SS is VgrG, which forms a phage-like tail needle structure together with the Hcp protein. This structure facilitates the injection of effector molecules into adjacent target cells through a conduit formed by PAAR (Proline-Alanine-Alanine-Arginine) proteins [11,12,13]. The VgrG protein is located at the tip of the T6SS pipeline [14]. Its structure comprises a variable active region, a gp5 region, and a gp27 region, which are similar to the tail spike of a T4 bacteriophage. This structure is responsible for penetrating the cell membrane [15], allowing the effector protein to reach specific cellular regions where it interacts with target molecules, thereby affecting host cell functions [16].

Bacterial toxins play a vital role in enhancing pathogenicity and triggering host immune responses. Lipopolysaccharide (LPS) and bacterial lipoprotein are key components of gram-negative bacteria. LPS is a major component of the outer membrane of gram-negative bacteria and has been shown to activate inflammatory pathways and induce host immune responses, and bacterial lipoproteins also promote pathogenesis by interacting with host receptors and modulating immune responses [17,18]. The *VgrG2* is a key factor in bacterial virulence and plays an important role in the interaction between bacteria and host cells. The mTOR signaling pathway is a critical intracellular signaling pathway that plays a role in a variety of physiological and pathological processes and has a significant regulatory role in the initiation and progression of autophagy [19]. Surviladze et al. discovered that human papillomavirus type 16 inhibits autophagy by activating the mTOR pathway, which facilitates its entry into host cells [20]. The mTOR pathway is also involved in autophagy induced by coxsackievirus B3 (CVB3) in HeLa cells [21]. Additionally, Yuying et al. demonstrated that Vibrio parahaemolyticus *VgrG2* can induce autophagy in macrophages [22]. However, the role of *E. coli VgrG2* in the mTOR signaling pathway and autophagy is still unclear.

In this study, we used CRISPR/Cas9 combined with Red homologous recombination to achieve large fragment deletions of the *VgrG2* virulence gene in wild-type *E. coli* and explored the effect of the *VgrG2* gene on the mTOR signaling pathway. Our analysis of key genes in the mTOR pathway revealed that *E. coli VgrG2* can activate the mTOR pathway and upregulate autophagy marker signals. These findings provide insights into the cellular damage mechanisms associated with *E. coli VgrG2* and further elucidate the pathogenic mechanisms underlying *E. coli*-induced host damage.

## 2. Materials and Methods

### 2.1. Cells, Strains and Plasmids

IPEC-J2 cells, derived from porcine immortalized small intestinal epithelial cells (Guangzhou Jennio Biotech, Guangzhou, China). The cells were cultured in DMEM supplemented with a 10% fetal bovine serum (FBS), penicillin, and streptomycin at 37 °C in a 5% CO_2_ incubator.

The eighty-three wild-type *E. coli* strains used in this experiment were isolated from the feces of diarrheal piglets at the Shaba Pig Farm in Lufeng County, Chuxiong Autonomous Prefecture, Yunnan Province, China. All strains were cultured in an LB medium at 37 °C.

Plasmids pTargetF (MC_0000012) with spectinomycin resistance and pCas (MC_0000011) with kanamycin resistance were obtained from Molecular Cloud.

### 2.2. PCR Detection of the Virulence Gene VgrG2

Based on the *E. coli VgrG2* gene sequence from GenBank (Accession No. CP006632.1), amplification primers were designed using the NCBI online tool: F: 5′-CGGATTACGTTTCACGCTGG-3′ and R: 5′-TTCATTAAACCCGCTGCCCT-3′. Genomic DNA was extracted from *E. coli* using a bacterial genomic DNA extraction kit (Tiangen Biochemical Technology, Beijing, China), and strains containing the *VgrG2* gene were identified by PCR with the designed *VgrG2*-F/R primers.

### 2.3. Resistance Screening

Strains carrying the *VgrG2* gene were inoculated in the LB medium until an optical density (OD_600_) of 0.6 was reached. Subsequently, 2 μL of this culture was transferred to 10 mL of the LB medium containing 50 mg/L kanamycin and mixed thoroughly. The OD_600_ was measured initially and again after 12 h of incubation at 37 °C with shaking at 160 rpm. Using a similar method, *E. coli* strains sensitive to 50 mg/L of both spectinomycin and kanamycin were further screened.

### 2.4. Construction of Knockout Vectors

The sgRNA primer sequence for *VgrG2* was designed using an online tool (http://spot.colorado.edu/~slin/cas9.html, accessed on 17 February 2025) as follows: sgRNA-*VgrG2*-SpeI-F: 5′-CTAGT*CCTGCCACCGGACGCGTTT*GGTTTTAGAGCTAGAAATAGC-3′ and sgRNA-*VgrG2*-R: 5′-GATGATCTTGCTTCATCTAGAGAATTCAAAAAAAGCACC-3′. The underlined bases represent the cleavage sites for SpeI, and the italicized bases indicate the overlapping sequences used to link the donor sequence via overlapping PCR. The designed sgRNA sequences were synthesized by a commercial company. The pTargetF plasmid was used as the template to amplify sgRNA-*VgrG2*.

To facilitate bacterial gene repair following cleavage, a homologous recombination template is required for targeted repair. Wild-type *E. coli* DNA as a template, and primers Arm-up-F/Arm-up-R and Arm-down-F/Arm-down-SalI-R, were used to amplify the upstream and downstream homology arms of the *VgrG2* knockout fragment, constructing the repair templates labeled L-Donor and R-Donor. The L-Donor and R-Donor were then ligated via overlap PCR. Subsequently, using this ligated donor and sgRNA-*VgrG2* as templates, and sgRNA-*VgrG2*-SpeI-F and R-Donor-SalI-R2 as primers, the final ligation was performed using overlap PCR, resulting in the product sgRNA-*VgrG2*-donor. Primer Sequences: L1: 5′-TGAAGCAAGATCATCCGGGC-3′; L2: 5′-GTGAGTCGACATCTGGCGTT-3′; R1: 5′-CAGATGTCGACTCACTAAAGTATGGCGGTCCATTGTC-3′; and R2: 5′-TCGACTGTTGGTCGCCAGGTAAAGA-3′. The underlined bases indicate the SalI restriction sites.

The ligated sgRNA-*VgrG2*-donor and pTargetF plasmids were treated with SalI and SpeI endonucleases to generate compatible ends. The pTargetF plasmid was then ligated with T4 ligase and sgRNA-*VgrG2*-donor. The ligation product was electroporated into *E. coli* DH5α, plated on LB agar containing spectinomycin, and incubated overnight at 37 °C. The following day, positive clones were screened, and plasmid DNA was extracted from monoclonal colonies. PCR identification was performed using primers pTargetF-JD-F and R. The products that matched the expected size of the target fragment were sent for sequencing verification. The validated vector was named pTargetF-sgRNA-*VgrG2*-donor. The identification primer sequences were as follows: F: 5′-ATTACCGCCTTTGAGTGAGC-3′ and R: 5′-GGATAACAGGGTAATAGATC-3′.

### 2.5. CRISPR/Cas9 Gene Editing Strategy for E. coli

To facilitate transfection, wild-type *E. coli* was first prepared as electrocompetent cells. The pCas plasmid was then electroporated into these cells and plated on LB agar containing kanamycin to select the positive clones. PCR identification was performed on the selected clones. The strains containing the pCas plasmid were made competent again, during which arabinose was added to induce the expression of the λ-Red protein from the pCas plasmid. The constructed vector, PTargetF-sgRNA-*VgrG2*-donor, was electroporated into *E. coli* containing the pCas plasmid and plated on LB agar containing both kanamycin and spectinomycin for selection. Monoclonal colonies were picked and identified by PCR using Δ*VgrG2*-JD-F/R primers, followed by sequencing. The primer sequences for Δ*VgrG2*-JD-F/R were as follows: F: 5′-TGCACATTATTGGAGGGGCA-3′ and R: 5′-TCCGTCACGCCGGTGATTT-3′.

### 2.6. Determination of the Growth Curve and Relative Cell Growth Rate

After incubating the target strain overnight, 200 µL of the culture medium is inoculated into 250 mL of the LB medium. The OD_600_ of the cultured strain is measured every 2 h for 24 h using a spectrophotometer. The growth curve of *E. coli* is plotted with time on the *x*-axis and mean OD_600_ on the *y*-axis. IPEC-J2 cells are infected with *E. coli*-WT and *E. coli* ∆*VgrG2*, and the relative growth rate of IPEC-J2 cells over the same 24 h period is assessed, with the blank group exhibiting a growth rate of 100%.

### 2.7. Cell Infection Experiments

To investigate the cell damage mechanism of *E. coli VgrG2*, 2 × 10^6^ IPEC-J2 cells were seeded into 6-well plates. Each well was supplemented with 2 mL of a cell culture medium containing 1 mL of the *E. coli* solution (OD_600_ = 0.6). The experimental setup included four groups: a blank group (control), *E. coli*-WT infection group, *E. coli∆VgrG2* infection group, and a rapamycin treatment group. Following infection, cells and cell supernatants were collected at 0.5 h, 3 h, 6 h, 9 h, 12 h, and 24 h for further analysis.

### 2.8. RT-qPCR Detection

The total RNA was extracted from cultured cells using the RNAiso Plus reagent (Takara, Dalian, China). Genomic DNA (gDNA) contamination was eliminated, and complementary DNA (cDNA) was synthesized using the PrimeScript RT Master Mix Kit with gDNA Eraser Kit (Takara, Dalian, China). Real-time PCR assays were conducted on a Bio-Rad CFX PCR instrument (Hercules, CA, USA) using TB Green Premix Ex Taq™ II. The mRNA expression levels of the *mTOR*, *ULK1*, *Beclin-1*, *Atg12*, *Atg5*, *Atg3*, *P62*, and *LC3* genes, which are associated with the mTOR signaling pathway, were normalized using the ΔΔCt method with β-actin as the reference gene. The primer sequences are provided in Table S1.

### 2.9. Cellular Immunofluorescence Detection

Pre-treated IPEC-J2 cells were fixed onto glass coverslips with 4% paraformaldehyde for 15 min, followed by permeabilization with 0.1% Triton X-100 for 10 min. After permeabilization, the cells were blocked with 5% bovine serum albumin (BSA) for 1 h. Specific primary antibodies (LC3-II Antibody, Proteintech Group, Inc., Wuhan, China, Cat. No. 14600-1-AP) were incubated with the cells overnight at 4 °C. Following incubation with the primary antibodies, the cells were washed three times with PBS and then incubated with the corresponding fluorescently labeled secondary antibody (Goat anti-rabbit IgG) for 1 h at room temperature in the dark. The cells were then stained with DAPI for 5 min. Fluorescent images of the stained cells were visualized using a fluorescence microscope (Olympus Corporation, Tokyo, Japan), and a quantitative analysis of the images was performed using ImageJ 1.8 software.

### 2.10. Statistical Analysis

Statistical analysis was based on a minimum of three biological replicates. Data were expressed as the mean ± SD and analyzed using either Student’s *t*-test or two-way ANOVA. Significance was defined as *p*-values less than 0.05.

## 3. Results

### 3.1. Identification and Resistance Screening of the Virulence Gene VgrG2 in Pathogenic E. coli

**Figure 1:**
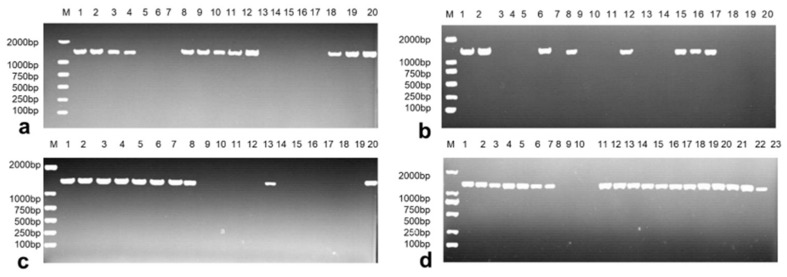
Detection of the *E. coli VgrG2* gene. (**a**–**d**). A total of 50 *E. coli* strains containing the *VgrG2* gene were screened via PCR. A total of 83 wild-type *E. coli* strains were isolated. Lanes 1–20 in (**a**–**c**) represent strains 1–20, 21–41, and 42–61, respectively, and lanes 1–23 in (**d**) represent strains 62–83. (M: 2000 bp DNA marker and lanes 1–23 represent the experimental strains. The target fragment size is 1435 bp).

A total of 83 wild-type *E. coli* strains were isolated, and PCR amplification revealed that 50 strains contained the *VgrG2* gene (Figure 1a–d). These 50 *E. coli* strains were subsequently inoculated into the LB medium containing kanamycin (50 mg/L). Of these, three strains were found to be susceptible to kanamycin, representing 6% (3/50) of the total. The three kanamycin-susceptible strains were then inoculated into an LB medium containing spectinomycin (50 mg/L), and only one strain was sensitive to both kanamycin and spectinomycin, resulting in a dual-susceptibility rate of 33.33% (1/3). This dual-sensitive strain was selected as the target strain for *VgrG2* gene knockout.

### 3.2. Construction of the E. coli-VgrG2 Targeting Vector pTargetF-sgRNA-VgrG2-Donor

**Figure 2:**
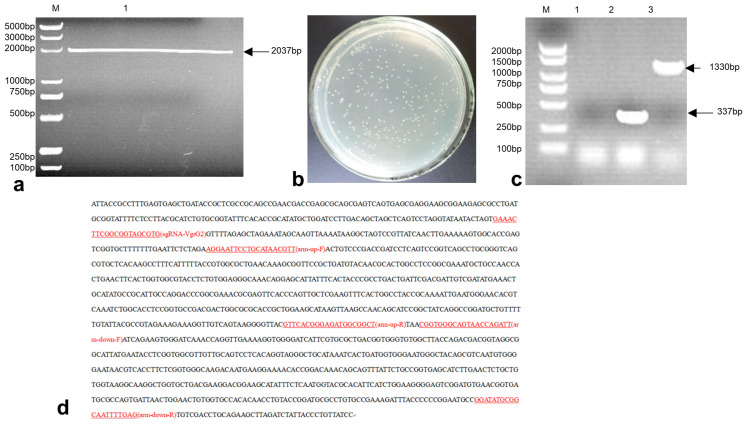
A construction of the recombinant plasmid. (**a**) Double digestion of the pTargetF plasmid with SalI and SpeI (M: 2000 bp DNA marker, lane 1: pTargetF plasmid double digested with SalI and SpeI); (**b**) colonies on the agar plate following transformation with the ligation product; (**c**) PCR verification of the pTargetF-*sgRNA-VgrG2*-donor plasmid construction (M: 2000 bp DNA marker, lane 1: negative control, lane 2: pTargetF plasmid, lane 3: pTargetF-*sgRNA-VgrG2*-donor plasmid); and (**d**) sequencing results of the PTargetF*-sgRNA-VgrG2*-donor plasmid.

The pTargetF plasmid was linearized by double digestion with SalI and SpeI enzymes, resulting in a product size of 2037 bp (Figure 2a). The sgRNA-*VgrG2*-donor was then ligated with the digested plasmid. The ligation product was electroporated into *E. coli* DH5α. The transformed bacteria were plated on LB plates containing spectinomycin and incubated overnight at 37 °C (Figure 2b). Single colonies were subsequently picked for PCR verification. A successful construction was indicated by a PCR product size of 1330 bp, while an unsuccessful attempt produced a product size of 337 bp (Figure 2c). Sequencing results were consistent with the expected sequence of the inserted sgRNA-*VgrG2*-donor (Figure 2d), confirming the successful construction of the pTargetF-sgRNA*-VgrG2*-donor plasmid.

### 3.3. Construction of the E. coli VgrG2 Virulence Gene Deletion Strain

**Figure 3:**
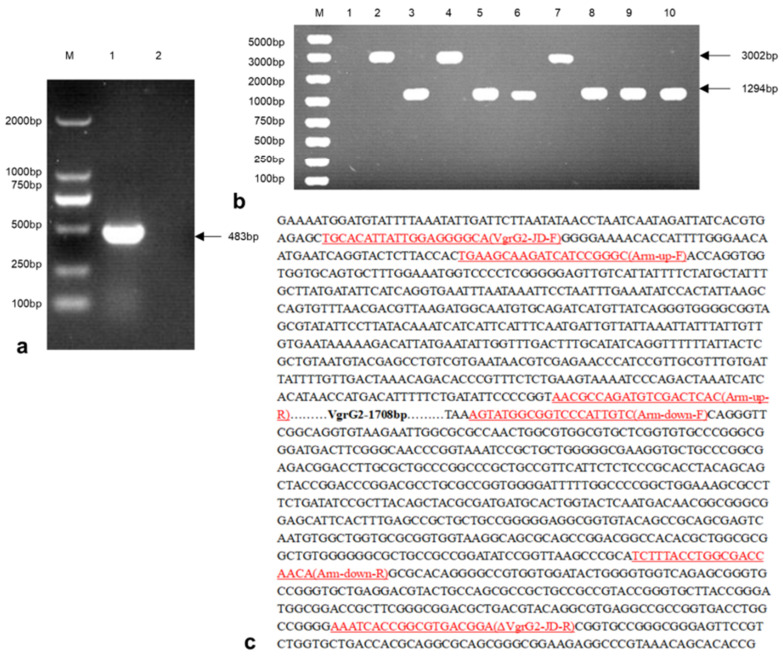
A construction of *E. coli* ∆*VgrG2* strains. (**a**) PCR verification of the PCas plasmid transformation results (M: 2000 bp DNA marker, lane 1: PCas plasmid transformed bacterial colonies, lane 2: negative control). (**b**) PCR verification of the *E. coli VgrG2* gene knockout strain construction (M: 5000 bp DNA marker, lane 1: negative control, lane 2: parent strains, lanes 3–10: experimental strains). (**c**) Sequencing results of the *E. coli VgrG2* gene knockout strain construction.

The PCas plasmid was electroporated into competent cells prepared from wild-type *E. coli.* Positive transformants were selected and subjected to PCR amplification, yielding a product size of 483 bp (Figure 3a). The pTargetF-sgRNA-*VgrG2*-donor plasmid was then electroporated into wild-type *E. coli* containing the PCas plasmid. Single colonies were picked for PCR analysis. A successful knockout was indicated by a PCR product size of 1294 bp, whereas an unsuccessful attempt produced a product size of 3002 bp (Figure 3b). Sequencing results confirmed the successful construction of the *VgrG2* gene deletion strain, which exhibited a total deletion of 1708 bp (Figure 3c).

### 3.4. The Effect of the VgrG2 Virulence Gene Deletion on the E. coli Growth Rate

**Figure 4:**
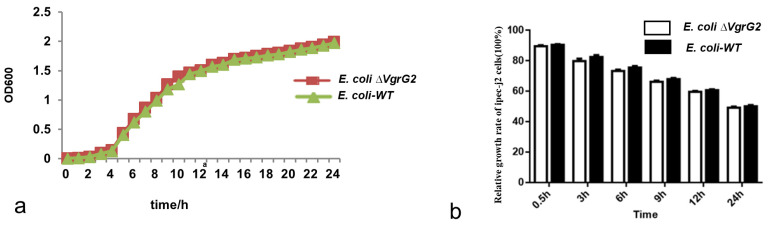
The effect of *VgrG2* gene knockout on strain and cell growth. (**a**) Growth curves of *E. coli-WT* and *E. coli* ∆*VgrG2*. (**b**) Relative growth rates of cells in the *E. coli-WT* and *E. coli* ∆*VgrG2* groups.

*E. coli WT* and *E. coli* ∆*VgrG2* strains were inoculated into a liquid culture medium and incubated with shaking, with the optical density recorded at different time points. Growth curves were plotted with culture time on the *x*-axis and optical density on the *y*-axis. The results showed that the growth trends of the strains were similar before and after the *VgrG2* gene knockout (Figure 4a), indicating that the deletion of the *VgrG2* virulence gene did not affect the growth activity of *E. coli*.

### 3.5. The Effect of E. coli-VgrG2 on IPEC-J2 Cell Growth

*E. coli-WT* and *E. coli* ∆*VgrG2* were used to infect IPEC-J2 cells. The relative growth rates of IPEC-J2 cells were not significantly affected by the *VgrG2* deletion at any of the measurement time points (Figure 4b).

### 3.6. E. coli VgrG2 Activates the mTOR Signaling Pathway in IPEC-J2 Cells

**Figure 5:**
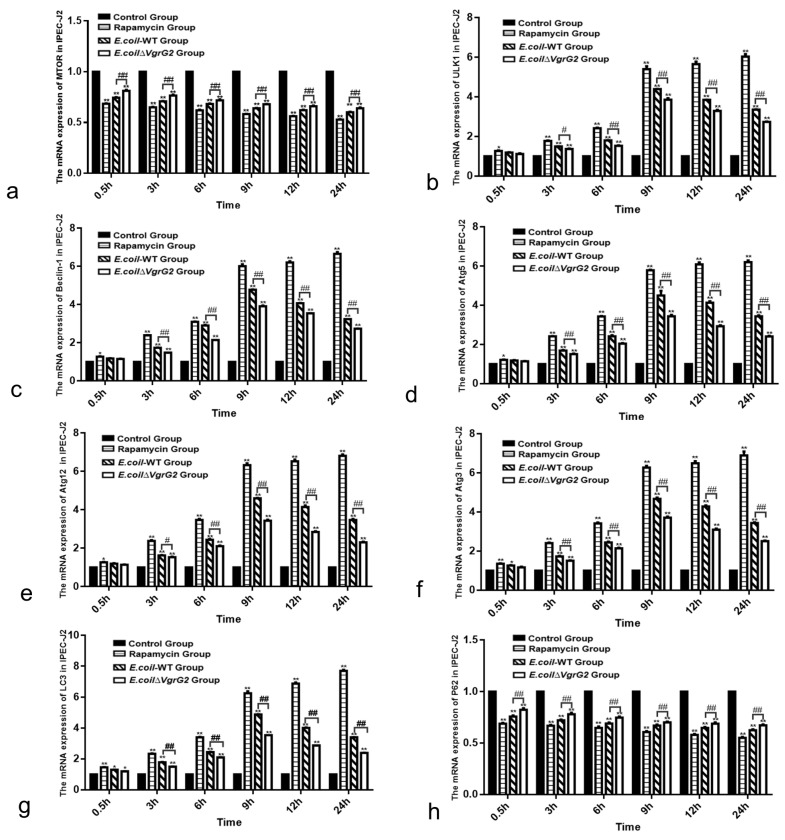
Detection of the expression levels of key genes in the mTOR signaling pathway at various time points using qPCR. (**a**) Relative expression levels of the *mTOR* gene in IPEC-J2 cells for each group at different time points. (**b**) Relative expression levels of the *ULK1* gene in IPEC-J2 cells for each group at different time points. (**c**) Relative expression levels of the *Beclin-1* gene in IPEC-J2 cells for each group at different time points. (**d**) Relative expression levels of the Atg5 gene in IPEC-J2 cells for each group at different time points. (**e**) Relative expression levels of the *Atg12* gene in IPEC-J2 cells for each group at different time points. (**f**) Relative expression levels of the *Atg3* gene in IPEC-J2 cells for each group at different time points. (**g**) Relative expression levels of the *LC3* gene in IPEC-J2 cells for each group at different time points. (**h**) Relative expression levels of the *P62* gene in IPEC-J2 cells for each group at different time points. All data are shown as the mean ± SD. Statistical analysis was performed using at least three biological replicates, analyzed using either Student’s *t*-test or two-way ANOVA. Significant differences compared to the control group: ** *p* < 0.01, * p < 0.05. Significant differences between the *E. coli-WT* group and the *E. coli* ∆*VgrG2* group: ## *p* < 0.01, # *p* < 0.05.

Programmed cell death is a significant mode of cell death, with autophagy representing one mechanism of this process. It has been established that mTOR promotes anabolism while inhibiting the induction of autophagy [23]. To further investigate the impact of *VgrG2* on the mTOR signaling pathway, we assessed the mRNA levels of key genes involved in this pathway at various time points following *E. coli* infection. The results demonstrated that the expression of autophagy-related genes *ULK1*, *Beclin-1*, *Atg5*, *Atg12*, *Atg3,* and *LC3* was up-regulated in IPEC-J2 cells (Figure 5b–g), whereas the expression of *mTOR* and *P62* was down-regulated (Figure 5a,h), an effect that was diminished in *E. coli* ∆*VgrG2*-infected cells. Collectively, these findings suggest that *E. coli* infection activates the mTOR signaling pathway in IPEC-J2 cells, with *VgrG2* enhancing this activation.

### 3.7. The Effect of E. coli VgrG2 on the Expression of the Autophagy Marker Protein LC3-II

**Figure 6:**
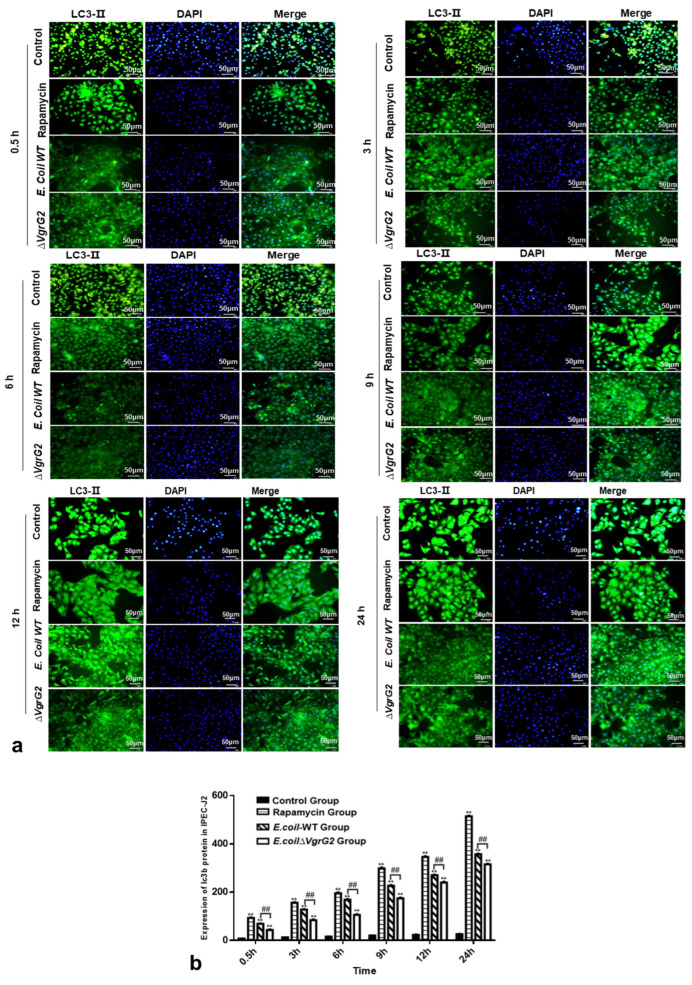
(**a**) Immunofluorescence staining to determine the expression of the LC3-II protein in IPEC-J2 cells for each group at various time points (400×) (where DAPI is the nuclear staining image, LC3-II is shown in green fluorescence, and Merge is the overlay of the first two images). (**b**) Total optical density sum values of the LC3-II protein expression in IPEC-J2 cells for each group at different time points. All data are shown as the mean ± SD. Statistical analysis was performed using at least three biological replicates, analyzed using either Student’s *t*-test or two-way ANOVA. Significant differences compared to the control group: ** *p* < 0.01. Significant differences between the *E. coli-WT* group and the *E. coli* ∆*VgrG2* group: ## *p* < 0.01.

LC3-II is a well-established marker of autophagy, where cytoplasmic proteins and organelles are sequestered by autophagosomes, with LC3-II being recruited to the autophagosome membrane during this process [24]. To assess the effect of *E. coli VgrG2* on LC3-II expression, we performed an immunofluorescence analysis in IPEC-J2 cells infected with either *E. coli*-WT or *E. coli ∆VgrG2*. Fluorescence microscopy revealed that LC3-II was recruited to the autophagosomal membrane, forming distinct fluorescent spots. A quantitative analysis of LC3-II fluorescence indicated an increase in the fluorescence intensity over time in infected IPEC-J2 cells, with *E. coli VgrG2* significantly enhancing LC3-II fluorescence (Figure 6a,b). In summary, our findings demonstrate that *E. coli* infection upregulates autophagy marker signals in IPEC-J2 cells and that *E. coli VgrG2* further promotes the production of these signals.

## 4. Discussion

In recent years, the rapid expansion of the pig farming industry has been accompanied by a rise in the incidence and drug resistance of *E. coli* infections, posing a significant threat to food safety and public health. Understanding the pathogenic mechanisms of *E. coli* is crucial for advancing the pig farming industry and safeguarding human health. Bacterial pathogens often use various mechanisms and pathways to infect hosts, cause tissue damage, and disrupt immune responses. Many of these pathogens rely on secretion systems to facilitate these processes. Our study focused on the *E. coli VgrG2* protein and found that among 83 pathogenic *E. coli* strains, 50 contained the *VgrG2* gene, resulting in a positive rate of 60.24%. This correlation suggests that *VgrG2* may play a role in the pathogenicity of *E. coli*. Resistance screening further revealed that only one strain out of the 83 was sensitive to both kanamycin and spectinomycin. This highlights the prevalent issue of drug resistance in *E. coli* within veterinary clinical settings and underscores the ease with which highly resistant and pathogenic strains can develop. This poses a considerable challenge to veterinary practice and public health.

The CRISPR/Cas9 gene editing system operates through the combined functions of sgRNA guidance, vector resistance labeling, and Cas9 specificity. Vector resistance labeling allows for the selection of positive clones on antibiotic-resistant plates, facilitating the verification of successful transfection. Guided by the sgRNA, the Cas9 protein precisely targets and cleaves a specific DNA sequence, creating a double-strand break. Research has demonstrated that CRISPR/Cas9 technology, with modifications to the pCas/pTargetF system, can be used for gene editing in *E. coli* strains CGMCC3705, K-12 MG1655, and DH5α, greatly reducing the time required for bacterial gene editing [25]. Although CRISPR/Cas9 technology in *E. coli* is well established, most of the research has been conducted with genetically engineered strains, with relatively few studies focusing on gene editing in wild-type *E. coli*. This presents challenges for studying gene functions in wild-type *E. coli* due to difficulties such as plasmid transformation, low transformation efficiency, and the more complex environment of wild-type strains compared to standard strains, which increases the risk of plasmid loss after transformation. In our study, we employed a two-plasmid system and prepared wild-type *E. coli* as competent cells for electroporation, facilitating the introduction of exogenous plasmids and improving transformation efficiency. We used LB plates containing kanamycin for the screening and identification of positive clones, which allowed for the rapid and accurate confirmation of strains transformed with the pCas plasmid. The constructed vector, pTargetF-sgRNA-*VgrG2*-donor, was electroporated into *E. coli* containing the pCas plasmid, and selection was performed on plates containing both kanamycin and spectinomycin. This dual-resistance screening enhanced the specificity and reliability of the selection process. The current results indicate a significant improvement in plasmid transfection efficiency, enabling successful CRISPR/Cas9-mediated gene editing in wild-type *E. coli*. This advancement addresses the challenges of genome editing in wild-type *E. coli* and offers new insights for studying gene function in wild-type strains.

*VgrG2* is considered a core component of the T6SS. In some strains, the T6SS gene cluster includes multiple *VgrG* genes with distinct roles. For example, Vibrio parahaemolyticus has been shown to induce autophagy in macrophages, with *VgrG2* deletion leading to reduced autophagy [22]. At physiological levels, autophagy helps cells manage various stress conditions, such as starvation, hypoxia, mitochondrial damage, and pathogen infection, thereby promoting cell survival. However, the negative aspects of autophagy are also becoming increasingly recognized; it can promote cell death by activating certain intracellular signaling pathways [26,27,28,29]. To explore the role of the *VgrG2* gene in wild-type *E. coli*, we used CRISPR/Cas9 technology to construct a ∆*VgrG2* strain and compared its growth with that of the wild-type (WT) strain. A growth curve analysis revealed that the growth trends of WT and ∆*VgrG2* strains were similar at all time points, indicating that *VgrG2* does not affect *E. coli* growth. We also investigated the impact of *VgrG2* on the mTOR signaling pathway by examining the transcription levels of key genes, including *mTOR*, *ULK1*, *Beclin-1*, *P62*, *LC3*, *Atg3*, *Atg5*, and *Atg12*, following infection, and assessed the expression of the autophagy marker protein LC3-II. Our results indicated that *E. coli* infection significantly modulated the expression of key genes in the mTOR signaling pathway, with *VgrG2* playing a crucial role in this process. LC3-II, a marker of autophagy, mediates membrane extension and fusion, leading to the formation of autophagosomes [30,31]. Rapamycin, an mTOR inhibitor, promotes autophagy [32]. After treating IPEC-J2 cells with rapamycin, we observed an increased LC3-II protein expression. Similarly, *E. coli* infection elevated LC3-II levels. However, in the *E. coli* ∆*VgrG2* infection group, we observed lower levels of LC3-II, suggesting that *VgrG2* likely promotes autophagy in IPEC-J2 cells, potentially by influencing the mTOR signaling pathway and upregulating LC3-II expression. While this study focused on the impact of *E. coli VgrG2* on key mTOR signaling pathway genes, the specific mechanisms by which *VgrG2* induces cellular autophagy warrant further investigation.

## Data Availability

All data can be obtained from the corresponding author.

## Supplementary Materials

The following supporting information can be downloaded at https://www.mdpi.com/article/10.3390/vetsci12030249/s1; Table S1: Primers used for PCR amplification.
