# Supplementary material for: The CRISPR/Cas9-Mediated Knockout of VgrG2 in Wild Pathogenic E. coli to Alleviate the Effects on Cell Damage and Autophagy
Source: Vet Sci. 2025 Mar 5;12(3):249. doi: 10.3390/vetsci12030249 (PMC11945575; doi:10.3390/vetsci12030249)
Supplement: Supplementary file 1 [file vetsci-12-00249-s001.zip › Table S1.pdf]

Supplementary Information

CRISPR/Cas9-mediated large fragment deletion of virulence genes in wild-type *E. coli* and its effects on the mTOR signaling pathway

Tian-ling Pan[1,†],Jin-long Cha[1,†], Hao Wang[2,†],Jing-Song Zhang[1],Jin-long Xiao[1],Jue Shen[1],Meng Zhou[2], Yue Li[1],Jin-zhi Ma[1] , Kai-yuan Zhao[1],Yong-kang Zhang[1],Peng Xiao[1*],Hong Gao[1*]

Table S1. Primers Used for PCR Amplification

Primers	Sequence 5′–3′(forward/reverse)	Annealing Temperature(℃)	Product size(bp)
VgrG2 -F	CGGATTACGTTTCACGCTGG	60	1435
VgrG2 -R	TTCATTAAACCCGCTGCCCT		
PCas-JD-F	TCCATTACCCGTGCGTTTGA	55	483
PCas-JD-R	CGGTAACGCAGATCGGATGA		
PtargetF-JD-F	ATTACCGCCTTTGAGTGAGC	55	337
PtargetF-JD-R	GGATAACAGGGTAATAGATC		
Arm-up-F	TGAAGCAAGATCATCCGGGC	60	503
Arm-up-R	GTGAGTCGACATCTGGCGTT		
Arm-down-F	CAGATGTCGACTCACTAAAGTATGGCGGTCCCATTGTC	62	492
Arm-down-salⅠ-R	**TCGAC**TGTTGGTCGCCAGGTAAAGA		
sgRNA- VgrG2-SpeⅠ-F	**CTAGT***CCTGCCACCGGACGCGTTTG*GTTTTAGAGCTAGAAATAGC	63	127
sgRNA- VgrG2-R	GATGATCTTGCTTCATCTAGAGAATTCAAAAAAAGCACC		
ΔVgrG2-JD-F	TGCACATTATTGGAGGGGCA	60	3002
ΔVgrG2-JD-R	TCCGTCACGCCGGTGATTT		

mTOR-F	GCCACTCTCTGACCCAGTTC	60	209
mTOR-R	GGTTATCCCAACCACGAGCA		
ULK1-F	CACCCACCCAGTACCAGACC	62	175
ULK1-R	ACTTGGGGAGATGGTGTGTAAG		
Beclin-1-F	AGGAGCTGCCGTTGTACTGTTCT	62	94
Beclin-1-R	TGCTGCACACAGTCCAGGAA		
Atg5-F	CCTGAAGATGGGGAAAGAAAGA	62	140
Atg5-R	TCTGTTGGTTGCGGGATG		
Atg12-F	TACGGAGGTCTCCCCAGAAA	60	177
Atg12-R	ATGGTTCGGGTTCGCTCTAC		
Atg3-F	CACGACTATGGTTGTTTGGCTATG	62	127
Atg3-R	GGTGGAAGGTGAGGGTGATTT		
LC3-F	AACGAAATTCCTGGTGCCTGA	60	90
LC3-R	AAGGCTTGGTTAGCATTGAGCTG		
P62-F	CAGGTGAACTCCAGTCTCTACA	60	102
P62-R	GGTACAATGCCGCTTCCTTC		
β-actin-F	TGCGGGACATCAAGGAGA	60	175
β-actin-R	AGGAAGGAGGGCGGAAGAG		
