# Supplementary figures and images for: The CRISPR/Cas9-Mediated Knockout of VgrG2 in Wild Pathogenic E. coli to Alleviate the Effects on Cell Damage and Autophagy
Source: Vet Sci. 2025 Mar 5;12(3):249. doi: 10.3390/vetsci12030249 (PMC11945575; doi:10.3390/vetsci12030249)

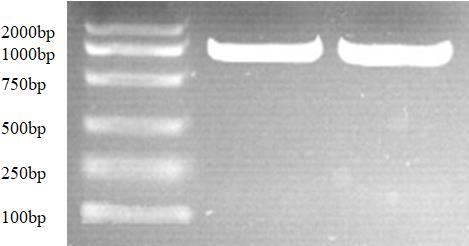

Supplement: Supplementary file 1 [file vetsci-12-00249-s001.zip › arm-up-down.jpg]

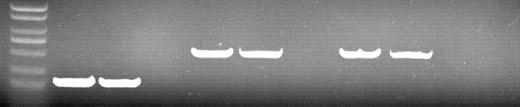

Supplement: Supplementary file 1 [file vetsci-12-00249-s001.zip › gRNA-VgrG2、arm-up、arm-down.jpg]

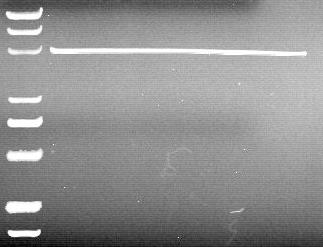

Supplement: Supplementary file 1 [file vetsci-12-00249-s001.zip › pTargetF plasmid Sal I, Spe I double restriction enzyme digestion.jpg]

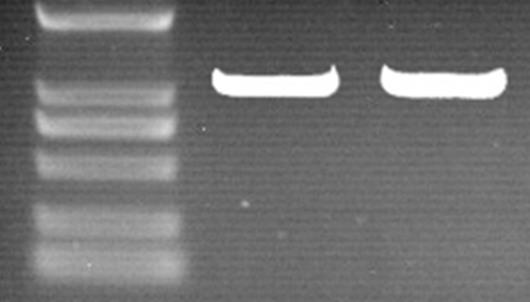

Supplement: Supplementary file 1 [file vetsci-12-00249-s001.zip › sgRNA-VgrG2-arm-up –down.jpg]

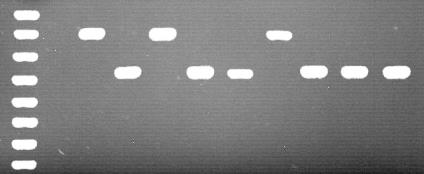

Supplement: Supplementary file 1 [file vetsci-12-00249-s001.zip › Verification of E. coli VgrG2 gene deletion strain.jpg]

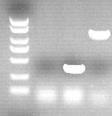

Supplement: Supplementary file 1 [file vetsci-12-00249-s001.zip › Verification of pTargetF recombinant plasmid construction.jpg]

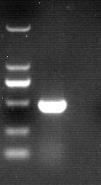

Supplement: Supplementary file 1 [file vetsci-12-00249-s001.zip › Verify pCas plasmid transformation results.jpg]

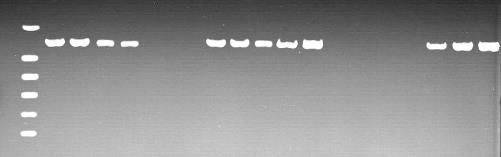

Supplement: Supplementary file 1 [file vetsci-12-00249-s001.zip › vgrg2. 1-20.jpg]

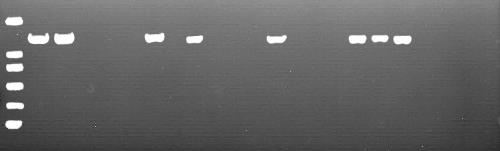

Supplement: Supplementary file 1 [file vetsci-12-00249-s001.zip › vgrg2. 21-41.jpg]

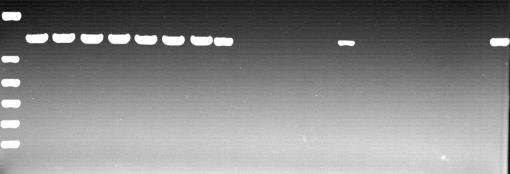

Supplement: Supplementary file 1 [file vetsci-12-00249-s001.zip › vgrg2. 42-61.jpg]

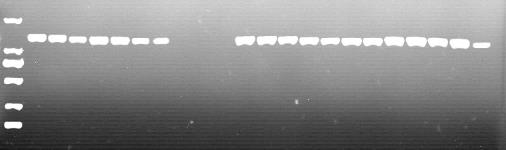

Supplement: Supplementary file 1 [file vetsci-12-00249-s001.zip › vgrg2. 62-83.jpg]
